# Prioritizing Pain Scale applications in the management of severe mental illness: literature update

**DOI:** 10.1192/j.eurpsy.2026.11487

**Published:** 2026-07-31

**Authors:** E. C. Ezema, E. U. Ezenagu, A. Meftah, B. Aribisala, S. Singh, T. Htet, T. Sultana, J. Beauchamp, T. Olupona

**Affiliations:** Psychiatry, One Brooklyn Health-Interfaith , Brooklyn, United States

## Abstract

**Introduction:**

It is increasingly evident that people who suffer from serious mental illness (SMI) have substantial physical ill-health, and they experience pain. Pain is well studied in depression but in people suffering from bipolar and psychotic disorders, it is understudied. The association between pain and psychosis is complex. People with schizophrenia have higher pain tolerance and thresholds. An estimated 30% of bipolar patients experience pain, which appears to have an impact on how the illness progresses. Considering the increased cardiometabolic burden associated with severe mental illness, along with the link between pain and cardiometabolic risk, pain treatment is critical. Pain is not routinely assessed in these patients. Therefore, it is essential to use pain assessment scales for the early identification and treatment of pain in these patients.

**Objectives:**

To determine the pain assessment scale commonly used in severe mental illness

**Methods:**

We conducted a review using search terms “Numerical Rating Scale (NRS) in pain management in severe mental illness,” “Visual Analog Scales (VAS) in severe mental illness,” “Wong-Baker Faces Pain Rating Scale (WBFPRS) in severe mental illness,” and “McGill Pain Questionnaire (MPQ) in severe mental illness,” The search was conducted on Google Scholar, PubMed, and the Bielefeld Academic Search Engine. It yielded the initial 13 articles we evaluated.

**Results:**

The Visual Analog Scale was found to be applied in 6 studies involving severe mental illness. It has also been adapted as a Psychological and Physical Pain VAS in psychiatric applications. A study revealed the application of WBFPRS in pain evaluation in severely mentally ill patients. The application of MPQ was reported in several psychiatric patients across 5 studies.

**Conclusions:**

Integrating routine pain assessment is essential in individuals with SMI because of the higher prevalence of comorbid physical conditions in them, and they are more susceptible to undertreated pain. Standardized pain scales must be appropriately selected to enhance care quality, improve clinical communication, and provide valuable data for creating a holistic treatment plan.

Prioritizing pain scale applications in the management of severe mental illness (SMI) offers significant benefits, but it requires addressing major challenges related to assessment, complexity, subjective interpretation, and adopting the appropriate scale.

**Disclosure of Interest:**

None Declared

